# Problematic mobile phone use among medical students and professionals: Its impact on sleep quality and depressive symptoms

**DOI:** 10.1192/j.eurpsy.2023.1388

**Published:** 2023-07-19

**Authors:** M. Abdellatif, H. Nefzi, O. Abidi, S. Blanji, R. Kamoun, M. Karoui, F. Ellouze

**Affiliations:** Psychiatry “G” department, Razi Hospital, manouba, Tunisia

## Abstract

**Introduction:**

Nowadays, the smartphone use is increasing, similar is the trend among medical students and professionals.

The problematic use of mobile phone has become a major public health concern as it may lead to its addiction and other disorders such as sleep disorders, depression and decreased life satisfaction.

**Objectives:**

This study aims to determine the association between mobile phone usage, Insomnia and depressive symptoms among medical students and medical professionals.

**Methods:**

We conducted a descriptive and analytical study among 40 medical students and doctors. They were asked to fill out an anonymous online survey.

Problematic Use of Mobile Phones (PUMP) scale was used to assess mobile phone usage. Insomnia Severity Index (ISI) was used to screen for insomnia and the Patient Health Questionnaire-9 (PHQ-9) to screen for depression.

**Results:**

The mean age of participants was 27,8 years, with a sex ratio of 1/3.

The mean of the duration of mobile phone use was 3.4 hours per day.

We found that 77% of participants spend most of their time on social media (Facebook, Instagram, Tik Tok) when using their mobile phones.

In our study, participants with higher problematic use of mobile phone (PUMP) score were significantly more likely to present symptoms of insomnia (p=0.031) and depression (p=0.023) according to ISI and PHQ-9 scales.

Furthermore, a significant association was found between the duration of mobile phone use, Insomnia and depression. In fact, the Odds Ratio (OR) of Insomnia was 1.66 in participants who used mobile phone more than 2 hours per day compared to those who used mobile phone < 2 hours per day.

Similarly, the OR of depressive symptoms was significantly increased with prolonged mobile phone use (>= 2 hours per day) compared to those who used it <2 hours per day (OR=3).

**Conclusions:**

Mobile phone problematic use is negatively related to sleep outcomes and depression symptoms. It is increasingly recognized as an important modifiable risk factor for mental health problems.

Prevention strategies including information, advice, sport and cultural activities are an essential need for all medical students and professionals to help them set limits for mobile phone use.

**Disclosure of Interest:**

None Declared

